# Age-related cognitive decline: a comparison between patients with type 1 bipolar disorder and healthy controls

**DOI:** 10.1192/j.eurpsy.2026.10703

**Published:** 2026-07-31

**Authors:** N. Smaoui, H. Sghaier, L. Triki, M. Maalej, R. Feki, I. Gassara, N. Charfi, M. Maalej, S. Omri, L. Zouari

**Affiliations:** 1 Psychiatry “C”, Hedi Chaker University Hospital; 2 Department of Functional Explorations, Habib Bourguiba University Hospital, Sfax, Tunisia

## Abstract

**Introduction:**

Cognitive decline occurs with normal aging and is often more pronounced in bipolar disorder type 1 (BD-1). Patterns of age-related cognitive impairment may differ between BD-1 patients and healthy individuals, with certain domains potentially more vulnerable to the effects of aging.

**Objectives:**

Analyze the relationship between age and cognitive performance in bipolar disorder type 1 (BD-1) compared with a control group, and to identify cognitive domains vulnerable to aging.

**Methods:**

This was a cross-sectional, case-control study. It was carried out at the “C” psychiatry outpatient unit at CHU Hedi Chaker in Sfax, Tunisia. Fifteen TB-1 patients and fifteen healthy controls were included. All participants were assessed by the Screen for Cognitive Impairment in Psychiatry (SCIP) scale for assessment of cognitive functions.

**Results:**

In cases, a significant negative correlation was observed between age and Verbal Learning Test-Immediate (VLT-I; ξ=-0.537∗) as well as Verbal Learning Test-Delayed (VLT-D; ξ=-0.556∗) scores, reflecting a decline in verbal learning abilities with advancing age. After multiple linear regression analysis, there was a negative correlation between total SCIP score and age (r= -0.4; p=0.012). For healthy controls, significant negative correlations were identified between age and SCIP total score (ξ=-0.516∗), VLTI (ξ=-0.547∗) and Processing Speed Test (PST; ξ=-0.719∗), indicating global cognitive impairment, particularly pronounced for processing speed. No significant associations were found for the Working Memory (WMT) or Verbal Fluency (VFT) subtests in either group (p > 0.05).

**Conclusions:**

This study revealed a differentiated age-related cognitive decline: impaired verbal learning in TB-1 patients and global impairments (SCIP score, processing speed) in controls. Targeted follow-up and longitudinal studies are needed to clarify these mechanisms.

**Disclosure of Interest:**

None Declared

